# Acute compartment syndrome following cardiovascular surgery: a rare and catastrophic complication highlighting the importance of early detection and intervention

**DOI:** 10.1186/s12872-025-04970-8

**Published:** 2025-07-18

**Authors:** Xianwen Hu, Youjun Pan, Zhiru Che, Yingya Cao, Xiaogan Jiang, Qiancheng Xu, Weihua Lu

**Affiliations:** 1 Department of Critical Care Medicine, The First Affiliated Hospital of Wannan Medical College (Yijishan Hospital of Wannan Medical College), Wuhu, 241000 Anhui China; 2 Anhui Provincial Clinical Research Center for Critical Respiratory Disease, Wuhu, 241000 Anhui China; 3 Department of Critical Care Medicine, Wuhu hospital, EastChina Normal University, Wuhu, 241000 Anhui China; 4 Perioperative Monitoring and Prognostic Technology Research and Development Center of Wuhu, Wuhu, 241000 Anhui China

**Keywords:** Acute compartment syndrome, Cardiovascular surgery, Femoral artery cannulation, Postoperative complications, Limb ischemia monitoring

## Abstract

**Background:**

Acute compartment syndrome (ACS) is a critical condition resulting from increased intra-compartmental pressure, causing tissue ischemia and necrosis. ACS following cardiovascular surgery is rare but catastrophic. Postoperative sedation and analgesia often obscure classic symptoms, delaying diagnosis. This underscores the importance of vigilance and early detection, particularly in high-risk scenarios such as prolonged extracorporeal circulation and femoral artery cannulation. Enhanced monitoring, including tissue oxygen saturation and transcutaneous oxygen pressure, may facilitate timely diagnosis.

**Case summary:**

We report a 56-year-old male who developed ACS after valve replacement surgery involving femoral artery cannulation for cardiopulmonary bypass. Approximately 12 h postoperatively, the patient exhibited severe lower limb swelling, mottling, and diminished dorsalis pedis pulse. Laboratory findings revealed elevated myoglobin and creatine kinase levels. Diagnosis was confirmed via clinical and ultrasound evaluation, prompting emergent fasciotomy. Postoperative management included wound care, renal replacement therapy, and skin flap reconstruction. At 6 months follow-up, the patient achieved complete functional recovery of the affected limb.

**Conclusion:**

ACS is a rare but severe complication of cardiovascular surgery. This case highlights the necessity for heightened vigilance, early recognition, and timely intervention to mitigate adverse outcomes. Further studies are needed to validate and establish standardized monitoring protocols and management strategies, including early use of distal perfusion techniques, to improve surgical safety and patient outcomes.

## Introduction

Acute compartment syndrome (ACS) is a clinical condition marked by an increase in intra-compartmental pressure, leading to progressive ischemia, hypoxia, and necrosis of the tissues within the affected fascial compartment. If left untreated, ACS can result in irreversible muscle necrosis, substantial loss of limb function, and permanent disability [1, 2]. The literature underscores that ACS can lead to severe complications, such as electrolyte imbalances, arrhythmias, acute renal failure, and systemic shock. In extreme cases, the amputation rate associated with ACS can reach as high as 31% [3, 4]. ACS also significantly increases the mortality risk in critically ill patients. In those undergoing extracorporeal membrane oxygenation (ECMO), lower limb ACS is associated with a substantially higher mortality rate, reaching up to 77% [5]. Even among non-ECMO patients, mortality rates still range from 16.7–20% [3, 4]. Furthermore, survivors of the acute phase may face long-term, debilitating functional impairment of the affected limb [3].

The etiological factors underlying ACS are multifactorial, with common causes including lower extremity fractures, soft tissue injuries, crush syndrome, and lower limb arterial embolism [1, 3, 6]. However, in more than 20% of cases, the precise cause remains elusive [6]. Among the less frequently reported etiologies, cardiovascular surgery has emerged as a potential but rare trigger of ACS. ACS following major cardiovascular surgery was first described by Pasic et al. in 1993 in a patient who underwent coronary artery bypass grafting (CABG) [7]. Since then, reports have been scarce, with most involving CABG [7–16]. Due to its low frequency, ACS in the context of cardiovascular surgery often goes undiagnosed, as highlighted by Etra et al., who stated that ACS following cardiovascular surgery is “easy to miss unless suspected“ [17]. Additionally, the postoperative management of cardiovascular surgery patients often involves the use of sedatives and analgesics to support cardiac recovery, which can obscure the classic signs of ACS, known as the 5Ps: pain out of proportion, pallor, paresthesia, paralysis, and pulselessness [18, 19]. As such, vigilant monitoring of lower limb function following cardiovascular surgery is essential for early detection and timely intervention.

We present a case of ACS that occurred following valve replacement surgery, utilizing femoral artery cannulation for establishing extracorporeal circulation. This case underscores a rare yet potentially catastrophic postoperative complication. It serves to emphasize the critical importance of raising awareness among clinicians regarding the risk of ACS after cardiovascular procedures and highlights the need for heightened vigilance, early recognition, and timely intervention to mitigate adverse outcomes.

## Case presentation

A 56-year-old male patient (59 kg, 168 cm) presented with a primary complaint of persistent chest tightness for over five months. The patient’s past medical history was significant for severe mitral regurgitation and moderate tricuspid regurgitation, as determined by transthoracic echocardiography (TTE) in 2021, which led to surgical mitral and tricuspid valve repair in that same year. The patient also had a 2-year history of type 2 diabetes mellitus, managed with oral empagliflozin, and no prior history of hypertension or hyperlipidemia. After a period of relative stability post-surgery, the patient began experiencing recurrent chest tightness in 2023, notably during episodes of restlessness. A follow-up TTE performed on August 23, 2023, demonstrated biatrial and left ventricular enlargement, moderate aortic valve regurgitation, moderate-to-severe mitral regurgitation, mild pulmonary hypertension (estimated pulmonary artery pressure: 45 mmHg), and moderate tricuspid regurgitation. Key measurements included a left ventricular diameter of 66 mm, a left atrial diameter of 56 mm, and an ejection fraction of 52%. On admission, his fasting blood glucose and serum creatinine levels were within normal limits. Due to recurrent valvular dysfunction, a comprehensive preoperative assessment was undertaken to evaluate cardiovascular risk factors and to exclude coronary artery or peripheral arterial disease. Electrocardiography revealed normal sinus rhythm with ST-T segment changes. Coronary angiography showed no significant coronary artery stenosis, while peripheral vascular ultrasound identified mild arteriosclerosis without significant luminal narrowing.

Following this comprehensive preoperative assessment, the patient was diagnosed with recurrent severe mitral regurgitation after mitral valve repair, moderate aortic valve regurgitation, and moderate tricuspid regurgitation. He was subsequently admitted to the Department of Cardiovascular Surgery for surgical intervention. On August 31, 2023, after careful preoperative planning, mitral valve replacement with a mechanical prosthesis (29#), aortic valve replacement with a mechanical prosthesis (21#), and tricuspid valve repair under cardiopulmonary bypass (CPB) were performed. Due to the short length of the ascending aorta, intraoperative decision-making led to the replacement of the aortic valve. CPB was established using a 24-Fr arterial cannula inserted into the right femoral artery. The procedure lasted approximately 8 h, during which the patient received 3 units of packed red blood cells and 600 mL of fresh frozen plasma. Throughout the surgery, hemodynamic stability was maintained with vasopressor support, including norepinephrine (0.17 µg/kg/min) and epinephrine (1.3 µg/kg/min). Further details of the surgical procedure and relevant perioperative parameters are presented in Table 1.

**Table 1:** Key events and laboratory values during surgery

Time (2023.8.31)	09:10	11:05	11:20	16:10	17:38
Procedure	Surgery start	Femoral artery cannulation	Initiation of CPB	Femoral artery decannulation	Surgery end
Mean Arterial Pressure (mmHg)	78	63	65	47	61
Body Temperature (°C)	26.6	36.3	34.5	36.0	35.9
Lactate (mmol/L)	1.1	--	2.6	8.7	10.7
pH	7.334	--	7.368	7.138	7.229
Blood Glucose (mmol/L)	4.5	--	5.6	10.4	11.2
Red blood cells(U)	--	--	--	3	--
Fresh frozen plasma(mL)	--	--	--	400	--
Epinephrine (µg/kg·min)	0	0.1	0.15	0.17	0.17
Norepinephrine (µg/kg·min)	0	0.25	0.7	1.0	1.3

*CPB* Cardiopulmonary Bypass

The patient was intubated and transferred to the intensive care unit (ICU). Sedation, analgesia, mechanical ventilation, and continued vasopressor therapy were provided to ensure hemodynamic stability in the ICU. On post-surgery day 1, marked swelling was observed in the right lower limb, with progressively increasing tension. Mottled changes were noted on the dorsum of the foot, and the pulse in the dorsal pedis artery was weaker than before. Additionally, urine output decreased progressively after the patient’s transfer to the ICU. Bedside ultrasound revealed localized muscle swelling in the right calf. Laboratory results showed elevated serum myoglobin (> 3997 ng/ml), creatinine (197 µmol/L), creatine kinase(3152 U/ml), and creatine kinase-MB (> 292 ng/ml). Compartment syndrome was diagnosed based on these findings.

**Fig. 1:**
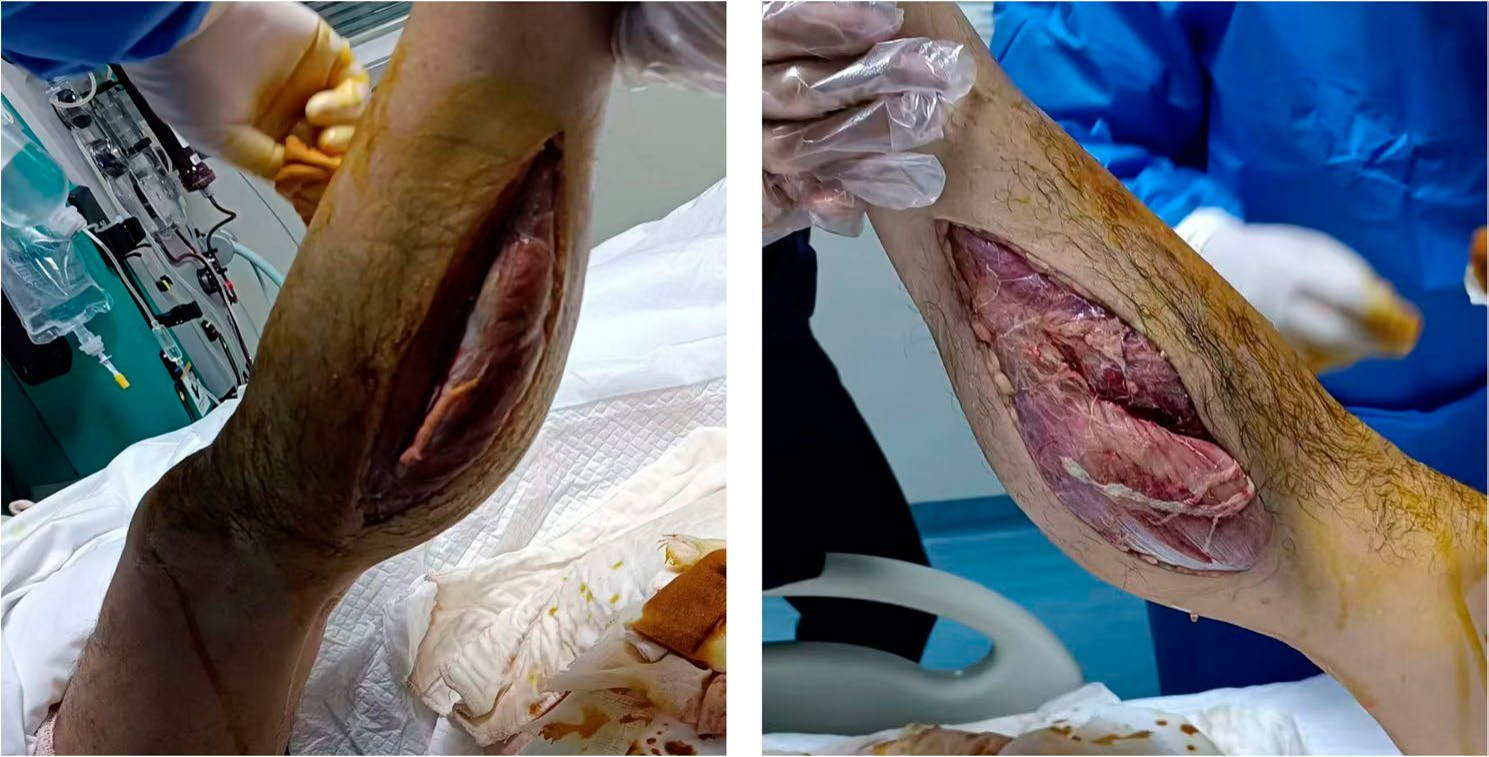
Fasciotomy of the Lower Limb in Acute Compartment Syndrome The image shows the incision made during the bedside fasciotomy procedure on the patient’s lower leg. Notable findings include deep muscle herniation and severe tissue edema, which were observed during the procedure, confirming the diagnosis of compartment syndrome

**Fig. 2:**
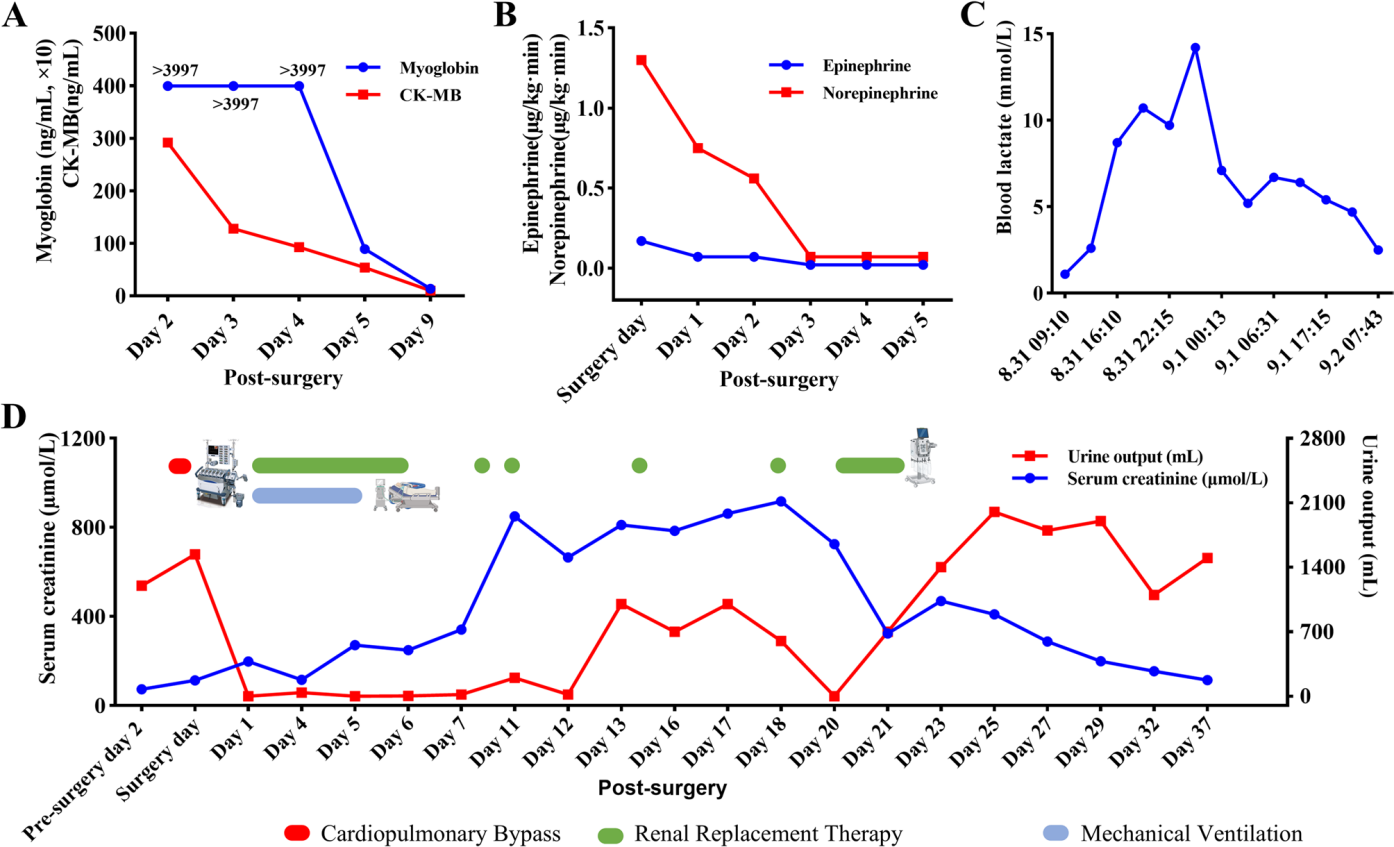
Dynamics of Biochemical Markers and Renal Parameters Panels A, B, and C show the trends of CK-MB, myoglobin, vasopressor dosage, and blood lactate levels in Patient A, with CK-MB and myoglobin gradually decreasing, while lactate rises initially and then declines. Panel D presents the serum creatinine levels (initial rise, plateau, followed by a decline) and urine output trends. The green line denotes the period of renal replacement therapy, and the red line indicates the duration of cardiopulmonary bypass, and the blue line denotes the duration of mechanical ventilation CK-MB: Creatine Kinase-MB

Given the clinical presentation and laboratory results, a bedside lower limb fasciotomy was performed (Fig. 1). During the procedure, deep muscle herniation was noted, along with severe tissue edema. After extensive dissection of the gastrocnemius, soleus, and posterior compartment, tissue relaxation was achieved, with notable improvement in the mottled appearance on the foot and restoration of pulsation in the dorsal pedis artery. During the ICU treatment period, the patient experienced hemodynamic instability, characterized by hypotension, arrhythmias, heart failure, and acute kidney injury. Additionally, metabolic disturbances, including electrolyte imbalances and acid-base disorders, were observed. Targeted interventions were implemented accordingly. Subsequent management focused on wound care as needed, renal replacement therapy (RRT) to maintain fluid-electrolyte and acid-base balance, nutritional support, and prophylactic antibiotic therapy to prevent infections (Fig. 2).

On post-surgery day 5, the patient’s vital signs stabilized, vasopressor support was discontinued, and respiratory function remained stable, allowing for successful weaning from mechanical ventilation. However, by post-surgery day 12, the wound continued to exhibit significant exudation. As a result, a right calf wound debridement and vacuum sealing drainage (VSD) was performed. Necrotic tissue was removed from the medial head of the gastrocnemius and the medial border of the soleus. After wound irrigation, the area was covered with two VSD dressings and sealed with a film dressing. On post-surgery day 21, wound exudation had significantly improved, and deep muscle viability was preserved. Renal replacement therapy was discontinued on the same day based on the criteria of a substantial increase in urine output (> 600 mL) and a marked decline in serum creatinine (323.8 µmol/L). However, some necrotic tissue remained. A skin flap reconstruction was performed for the right calf wound, during which necrotic tissue was excised and tension-relieving sutures were applied. At the 6-month follow-up, the patient’s lower limb function had completely recovered, and the wound had completely healed. The duration of RRT and mechanical ventilation treatment and the trends in relevant laboratory parameters (Myoglobin, creatine kinase-MB, blood lactate levels, vasopressor dosage, and renal function parameters) are presented in Fig. 2.

## Discussion

To date, only one case of ACS following valve replacement surgery has been documented; however, the patient also underwent a combined procedure involving CABG and aortic valve replacement [12]. This case highlights that ACS can occur even in valve replacement procedures performed under femoral artery cannulation with CPB, underscoring its potentially severe consequences.

In this case, ACS developed insidiously, presenting approximately 12 h postoperatively. Sedation and analgesia obscured the classical “5P” symptoms, delaying recognition until significant lower limb swelling, mottled foot skin, and weakened dorsalis pedis pulses became evident. A review of both English and Chinese journals reveals 11 reports concerning postoperative ACS following cardiac surgery, encompassing a total of 39 cases (Table 2). The time from onset to diagnosis ranged from immediately postoperative to up to 5 days after surgery, with the majority of cases occurring 12–48 h postoperatively. CPB duration varied from 0 min to 332.1 ± 79.7 min. Most patients experienced residual foot complications of varying severity, with only 15.4% (6/39) achieving complete limb recovery. These findings emphasize the importance of heightened vigilance during the 12–48-hour postoperative window. Regular monitoring of limb circumference and tension is essential for timely detection, as delayed diagnosis significantly worsens functional outcomes. Research indicates that ischemia lasting over 30 min can lead to nerve dysfunction, while irreversible tissue damage occurs after 12 h of ischemia. Furthermore, even after reperfusion, ischemia-reperfusion injury and tissue swelling can still trigger ACS [20, 21]. The estimated incidence of ACS following major cardiovascular surgery is approximately 0.003% [9], rendering it a rare but significant complication. While its etiology is multifactorial, the pathophysiology predominantly involves ischemia and hypoxia of the lower extremities during surgery. Key contributing mechanisms include:

**Table 2:** Postoperative acute compartment syndrome following cardiovascular surgeries: summary of published cases

Author	No.	Surgery	Time to diagnosis (postoperative)	CPB time (minutes)	Outcome
Pasic, 1993 [7]	2	CABG	1 day	Not reported	Dorsiflexion weakness, sensory loss
		CABG	8 h	Not reported	Paresthesia without muscular weakness
van den Wildenberg, 1996 [8]	3	CABG	1 day	63	Peroneal and posterior tibial neuropathy
		CABG	1 day	90	Above-knee limb loss
		CABG	40 h	60	Below-knee limb loss
Ted James, 2002 [9]	2	CABG	1 day	156	Foot drop
		CABG + IABP	2 days	51	Foot drop, decreased sensation
Vaidyanathan, 2006 [11]	1	Off-pump CABG	24 h	0	Foot drop
Papas, 2007 [12]	1	CABG + aortic valve replaced	2 days	Not reported	Minimal foot drop.
Aparna, 2010 [16]	1	CABG	4 days	94	Complete recovery
Al-Sarraf, 2010 [13]	1	CABG	24 h	57	Complete recovery
Mills, 2010 [14]	1	CABG	2 days	Not reported	Bilateral foot drop
Asgun, 2013 [15]	1	CABG	12 h	72	Foot drop and sensory loss
Te Kolste, 2015 [10]	5	CABG	2 days	167	Complete recovery
		CABG	1 day	151	Above-knee amputation
		CABG	1 day	83	Complete recovery
		CABG	5 days	189	Complete recovery
		Off-pump CABG + TAVR	2 days	0	Foot drop
Zhong, 2017 (Published in Chinese) [26]	21	14 CABG cases, Including: 1 case with ECMO support alone; 5 cases with IABP support alone; 8 cases with combined ECMO and IABP support.	Immediately (4 cases) 12 h (3 cases) Others: Not reported	129.9 ± 47.2	3 cases: relief without surgical intervention. 6 cases: Residual limb dysfunction. 9 cases: Fatal, including 3 cases with above-knee amputation. Others: Not reported.
		7 Type A dissection cases, Including: 2 cases with ECMO support alone		332.1 ± 79.7	

*CABG* Coronary artery bypass grafting, *CPB* Cardiopulmonary bypass, *IABP* Intra-aortic balloon pump, *ECMO* Extracorporeal membrane oxygenation, *TAVR* Transcatheter aortic valve replacement

### Femoral artery cannulation and arteriosclerosis

Femoral artery cannulation during CPB relies on the residual flow around the cannula to maintain distal perfusion, which is determined by the degree of arterial occlusion and the cannula-to-artery diameter ratio [22]. Evidence from VA-ECMO studies demonstrates that smaller arterial cannulas (< 17 F) significantly reduce ischemia risk. A meta-analysis by Marbach et al., comprising 22 studies and 2,869 patients, reported that cannulas < 17 F were associated with a lower risk of ischemia (OR = 0.40, 95% CI: 0.24–0.65, *P* < 0.001) [23]. Retrospective analyses have consistently identified cannula size as a critical determinant of ischemic complications, with larger cannulas, particularly those with a cannula-to-body surface area ratio > 11, increasing the risk of limb ischemia. Conversely, smaller cannulas (< 15 F) are associated with a reduced incidence of ischemia [24, 25].

### Low perfusion and prolonged CPB duration

Prolonged CPB has been established as a risk factor for ACS. Ischemia risk significantly increases when CPB duration exceeds 90 min [8, 9]. In a series from Chinese Anzhen Hospital, CABG patients had an average CPB time of 129.86 ± 47.23 min, whereas patients undergoing aortic replacement reported an average CPB time of 332.14 ± 79.74 min, both associated with increased ACS incidence [26]]. In the present case, CPB duration reached 290 min, far exceeding the 180 min threshold associated with prolonged perfusion-related complications [32]. In such scenarios, monitoring femoral artery pressures (< 50 mmHg) may guide DPC placement decisions.

### Impaired venous return

Impaired venous return has been proposed as a contributing factor to ACS. Early hypotheses linked ACS to saphenous vein harvesting and leg compression during CABG. However, reports from Zhang et al. found no cases of ACS solely attributable to saphenous vein harvesting. Intriguingly, 6 cases involved the contralateral limb [26]. Considering that over 800,000 CABG procedures are performed annually worldwide [33], the routine nature of vein harvesting suggests it is unlikely to be an independent cause of ACS but rather one of the factors that may exacerbate its occurrence.

### Monitoring, early detection, and prevention of limb ischemia process

**Fig. 3:**
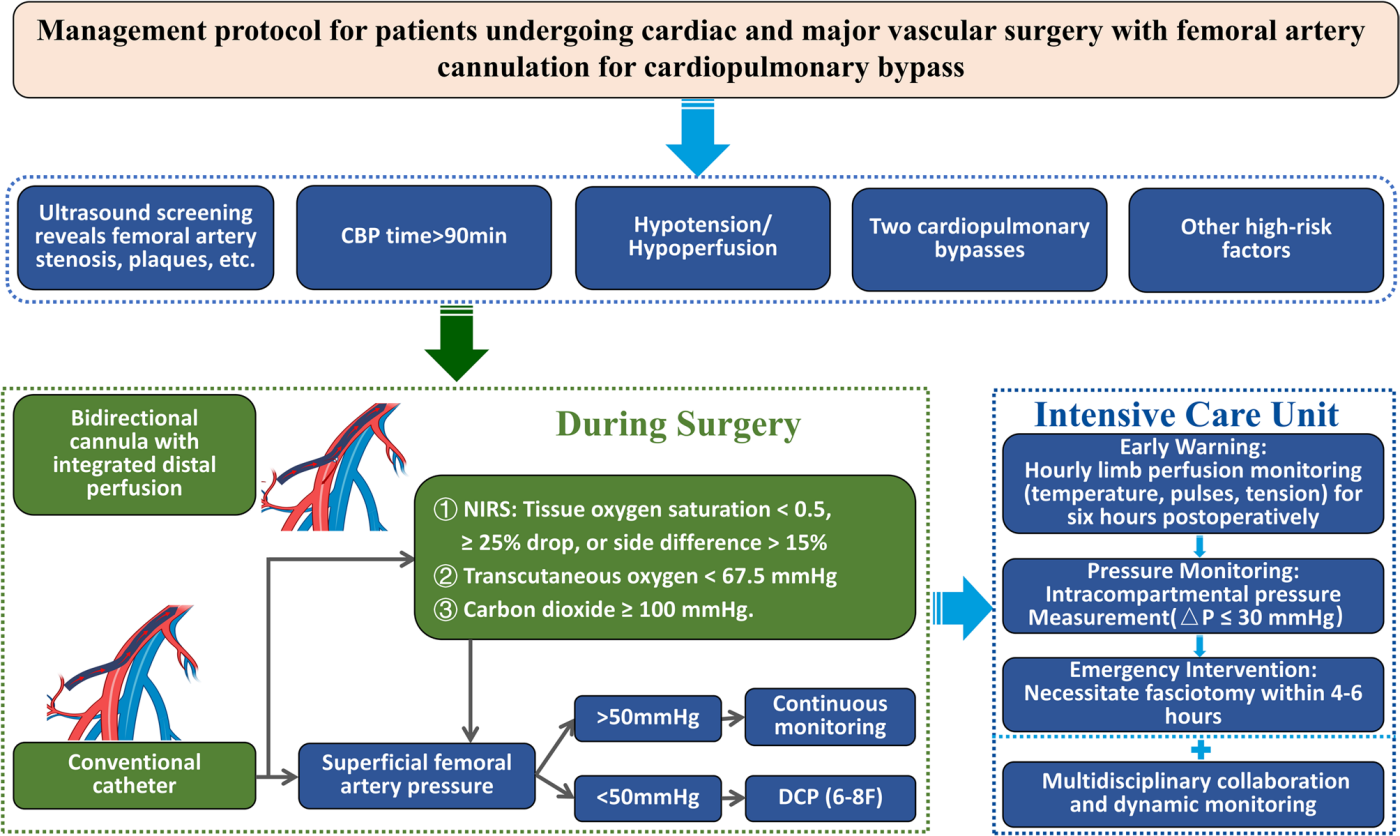
Proposed Workflow for Prevention and Management of Acute Compartment Syndrome in the Lower Limb This flowchart presents a structured approach to preventing and managing acute compartment syndrome. Key steps include femoral artery ultrasound evaluation, monitoring tissue perfusion parameters (e.g., oxygen saturation, transcutaneous oxygen pressure, and carbon dioxide pressure), and timely intervention with distal perfusion catheter placement based on ischemia thresholds. In the ICU, management follows the principles of Early Warning, Pressure Monitoring, and Emergency Intervention, ensuring prompt detection and timely treatment to optimize patient outcomes NIRS: Near-Infrared Spectroscopy; ICU: Intensive Care Unit; △P: Difference between the diastolic blood pressure and the compartment pressure

Given the rarity of ACS, the cost-effectiveness of universal preventive measures (e.g., placing DCP) is limited. Enhanced monitoring and timely intervention are thus critical. Muscle necrosis can occur within 3 h of ischemia, highlighting the urgency of early detection [34]. Near-infrared spectroscopy (NIRS), transcutaneous oxygen pressure (TcPO2), and transcutaneous carbon dioxide pressure (TcPCO2) offer effective tools for monitoring limb perfusion. NIRS achieves 100% sensitivity and 95% specificity for lower limb ischemia when tissue oxygen saturation falls below 0.50 or decreases by > 25% from baseline or a side-to-side difference > 15% [35, 36]. Similarly, TcPO2 < 40 mmHg or TcPCO2 ≥ 100 mmHg strongly suggests critical ischemia, with normalization after reperfusion [37, 38]. A meta-analysis identified TcPO2 ≤ 20 mmHg as a threshold for amputation [39].Therefore, to monitor and intervene in lower limb ischemia, the following process is proposed (Fig. 3): Ultrasound evaluation of the femoral artery is essential for detecting potential abnormalities, including stenosis, plaques, calcification, dissection, thrombosis, or aneurysms. In cases where abnormalities are identified, bidirectional cannula with integrated distal perfusion placement may be warranted. For patients at high risk of ischemia, such as those with a large conduit-to-artery diameter ratio, femoral artery sclerosis or plaques, prolonged extracorporeal circulation exceeding 90 min, sustained low perfusion indicated by a high VIS score, or secondary transfers, if a conventional catheter is placed, monitoring lower limb tissue perfusion is crucial. Key parameters for assessing lower limb tissue perfusion include tissue oxygen saturation, TcPO2, and TcPCO2. Tissue oxygen saturation levels should be monitored for values below 0.50, reductions exceeding 25% from baseline, or side-to-side differences greater than 15%. TcPO2 levels below 67.5 mmHg, and TcPCO2 levels of 100 mmHg or higher, are indicative of critical ischemia. These thresholds necessitate prompt evaluation and intervention to prevent further complications. If femoral artery pressure remains < 50 mmHg, immediate placement of a DPC is recommended. Conversely, if femoral artery pressure exceeds 50 mmHg, DPC placement may be deferred while other potential causes are investigated. Upon transfer to the ICU, postoperative limb perfusion should be assessed hourly within the first 6 h, incorporating clinical indicators (skin temperature, pulses, compartment tension) [40]. For suspected ACS cases (e.g., pain on passive stretching, abnormal compartment firmness), immediate intracompartmental pressure measurement is required, with a critical threshold of ΔP ≤ 30 mmHg [41, 42]. Confirmed ACS mandates fasciotomy within the 4–6-hour “golden window”, supported by multidisciplinary collaboration (e.g., vascular surgical intervention [43]) and dynamic monitoring of creatine kinase and hemodynamic parameters [44]. However, it is important to note that there is limited conclusive evidence supporting the widespread implementation. Therefore, these strategies should be considered as part of a broader approach, taking into account individual patient circumstances and the results of additional diagnostic tests. A more nuanced, case-specific approach is essential for optimizing patient care, as the management of ACS remains context-dependent.

### Limitations

First, due to the relatively low incidence of ACS in cardiovascular surgeries, reports based on single cases may not fully represent all related factors. Second, although enhanced monitoring methods such as NIRS and TcPO2 were mentioned, their universal applicability and standardized use require further validation. Additionally, early diagnosis of ACS still relies on clinical experience, and data from more multi-center studies were not included to further support the effectiveness of monitoring and prevention strategies. Therefore, future research should conduct larger-scale, multi-center clinical studies to verify these findings.

## Conclusion

This case highlights the possible risk of ACS following valve replacement surgery, particularly in relation to femoral artery cannulation. Prompt recognition, appropriate diagnosis, and multidisciplinary cooperation may contribute to effective management and improved patient outcomes. Enhanced postoperative monitoring could be important for high-risk patients. However, further studies are needed to better understand the mechanisms, risk factors, and optimal management strategies for postoperative ACS in order to inform clinical practice.

## Data Availability

No datasets were generated or analysed during the current study.

## Abbreviations

ACS: Acute Compartment Syndrome
CABG: Coronary Artery Bypass Grafting
CK-MB: Creatine Kinase-MB
CPB: Cardiopulmonary Bypass
RRT: Renal Replacement Therapy
DPC: Distal Perfusion Catheter
ECMO: Extracorporeal Membrane Oxygenation
ICU: Intensive Care Unit
NIRS: Near-Infrared Spectroscopy
TcPCO2: Transcutaneous Carbon Dioxide Pressure
TcPO2: Transcutaneous Oxygen Pressure
VSD: Vacuum Sealing Drainage
